# The Potential Predictive Role of Oxidative Stress and Antioxidant Parameters Regarding Mortality and the Type of Causative Agent in Sepsis

**DOI:** 10.7759/cureus.73456

**Published:** 2024-11-11

**Authors:** Milana Stanic, Sasa Dragic, Maja Travar, Snezana Uletilovic, Nebojsa Mandic-Kovacevic, Pedja Kovacevic

**Affiliations:** 1 Anesthesiology and Intensive Care, University Clinical Centre of the Republic of Srpska and Medical Faculty University of Banja Luka, Banja Luka, BIH; 2 Medical Intensive Care Unit, University Clinical Centre of the Republic of Srpska and Medical Faculty University of Banja Luka, Banja Luka, BIH; 3 Clinical Microbiology, University Clinical Centre of the Republic of Srpska and Medical Faculty University of Banja Luka, Banja Luka, BIH; 4 Medical Biochemistry and Chemistry, Centre for Biomedical Research, Medical Faculty University of Banja Luka, Banja Luka, BIH; 5 Pharmacy, Centre for Biomedical Research, Medical Faculty University of Banja Luka, Banja Luka, BIH

**Keywords:** antioxidants, outcome, oxidants, prediction, sepsis

## Abstract

Oxidative stress represents an imbalance between oxidants and antioxidants, with a predominance of oxidants leading to cellular and tissue damage. Given the limited number of studies showing the predictive value of oxidative stress factors regarding sepsis type, the objectives of this study emerged as follows: to determine whether pro-oxidant and antioxidant values could predictively differentiate between Gram-positive (GP) and Gram-negative (GN) sepsis. Additionally, the study sought to assess whether bacterial type impacts treatment outcomes in sepsis patients. This prospective, observational cohort longitudinal study included 87 patients diagnosed with sepsis according to the Third International Consensus on Sepsis and Septic Shock (Sepsis-3) criteria. Following the sepsis diagnosis, blood, urine, bronchoalveolar lavage (BAL), and swabs/punctures were sampled and microbiologically analyzed. Sampling was repeated 24 hours after the first collection. Based on the microbiological results, four groups of subjects were formed: the GP group included septic patients in whom one or more GP bacteria were isolated by microbiological analysis; the GN group included septic patients in whom one or more GN bacteria were isolated; the GP/GN group included septic patients with both GP and GN bacteria isolated; and the negative culture (NC) group included septic patients in whom no pathogenic microorganisms were detected by microbiological analysis. Additionally, after sepsis diagnosis, oxidative stress markers, i.e., thiobarbituric acid reactive substances (TBARS), nitrite ion radical (NO₂⁻), hydrogen peroxide (H₂O₂), superoxide ion radical (O₂⁻), and antioxidants: superoxide dismutase (SOD), catalase (CAT), and reduced glutathione (GSH) were determined from blood samples. Blood sampling was repeated 24 hours after the first collection. On comparing pro-oxidant values relative to the type of infection in septic patients, measured on the first and second days, no statistically significant differences were observed for the analyzed parameters, except for delta (Δ) O₂⁻. A statistically significant increase was noted in the GN group of septic patients (p = 0.02). On comparing antioxidant values relative to infection type in septic patients, measured on the first and second days, no statistically significant differences were found for the analyzed parameters. Based on the measured values of pro-oxidants and antioxidants in this study, it is evident that they do not have predictive significance for the final treatment outcome and the type of sepsis-causing pathogen. These results underscore the need for further research on the predictive role of oxidative stress in sepsis.

## Introduction

In the Third International Consensus on Sepsis and Septic Shock (Sepsis-3), sepsis was defined as life-threatening organ dysfunction caused by an inadequate host response to infection [1]. For clinical assessment of organ dysfunction, the Sequential Organ Failure Assessment (SOFA) score is used [2]. Sepsis is the leading cause of mortality in intensive care units [3,4]. The incidence of sepsis is rising worldwide, and it is one of the most common causes of death globally [5,6]. According to 2020 data, there were 48.9 million cases of sepsis and 11 million deaths associated with sepsis, representing 20% of all global deaths [7]. The pathogenesis of sepsis is still not fully understood, but one of the key phenomena within the inflammatory response is the cell’s inability to utilize oxygen and the excessive production of oxidants. Increased oxidant production, reduced availability, and/or increased consumption of antioxidants disrupt “redox homeostasis,” leading to damage to cellular lipids, proteins, and nucleic acids [8,9]. It is known that in septic patients, the parameters of oxidative stress outweigh those of antioxidants. The causative imbalance of proteins, lipids, and nucleic acids in septic patients results in cellular damage and impaired endothelial function, further deteriorating the glycocalyx and intercellular junctions and leading to increased vascular permeability, which is a fundamental aspect of sepsis progression [10]. Most studies that have monitored levels of oxidative stress and antioxidants were conducted over a single time period, very rarely as longitudinal studies, and most often at the very onset of sepsis. Most of them show a dominance of oxidants over antioxidants [11,12]. The mechanisms by which bacteria cause sepsis and septic shock are well known, as is the fact that the main causative agents are Gram-negative (GN) bacteria [13,14]. In the last 20 years, greater importance has been placed on Gram-positive (GP) bacteria as causative agents of sepsis, and the degree of inflammation associated with some compared to others is controversial [15-17].

Regarding the limited number of literature sources that demonstrate the predictive significance of oxidants and antioxidants regarding the type of sepsis (GN vs. GP), our study objectives have emerged as follows:

To determine whether the measured values of pro-oxidants and antioxidants can have predictive significance regarding the causative agents of sepsis (GN vs. GP).

To demonstrate whether different types of bacterial infections (GN vs. GP) can have predictive significance in the final outcome of critically ill patients with sepsis or septic shock.

## Materials and methods

Study design

In this prospective, observational cohort longitudinal study, 87 patients were included. The research was conducted at the Surgical Intensive Care Unit (SICU) and Medical Intensive Care Unit (MICU) of the University Clinical Center of the Republic of Srpska (UCC RS) from July 2021 to February 2023. Microbiological analyses were performed at the Department of Microbiology, UCC RS, and the Center for Biomedical Research, Faculty of Medicine, University of Banja Luka, Banja Luka, Bosnia and Herzegovina. These intensive care units serve as referral centers for the Republic of Srpska, covering a population of approximately 1 million inhabitants. It is important to highlight that Bosnia and Herzegovina is a post-war, transitional country that is geographically part of the Western Balkans. The study was approved by the Ethics Committee of the UCC RS (approval no. 01-19-181-2\21, dated 20.04.2021) and the Ethics Committee of the Faculty of Medicine, University of Banja Luka (certificate no. 18\4.12\21, dated 01.02.2021).

Patient selection

Patients for whom written consent has been obtained (either personally or from a legal representative), aged over 18 years, and diagnosed with sepsis according to the Sepsis-3 criteria (an acute increase in SOFA score of 2 or more points from baseline in patients with suspected infection) were included in the study. Patients with burns and a diagnosis of pancreatitis, those suffering from systemic inflammatory and autoimmune diseases, pregnant women, patients currently undergoing immunosuppressive therapy, patients who have received blood derivatives, and patients who are seropositive for the SARS-CoV-2 virus confirmed by standard polymerase chain reaction (PCR) testing, were excluded from the study. The exclusion criteria included a lack of increase in the SOFA score by 2 points after 72 hours of admission to the MICU and SICU, as well as patients who developed a SARS-CoV-2 infection during the study. Based on the microbiological results obtained, we identified four groups of septic patients: group I included GP septic patients in whom one or more GP bacteria were isolated by microbiological analysis; group II included GN septic patients in whom one or more GN bacteria were isolated by microbiological analysis; group III included polymicrobial group (GP/GN) patients in whom both GP and GN bacteria were isolated by microbiological analysis; and group IV included negative culture (NC) samples in which no pathogens were found through microbiological analysis. The observed patients were monitored for 28 days after being included in the study.

Study parameters

All patients included in the study were assigned a quick SOFA (qSOFA) score. For participants with a qSOFA score of 2 or 3 points, the SOFA score was determined daily for the next 72 hours until the diagnosis of sepsis was established. All scores were calculated using the electronic application MDCalc (www.mdcalc.com). The criteria from the Sepsis-3 were used to diagnose sepsis.

Microbiological sampling 

Blood was drawn from participants for blood cultures, and urine was collected for urine culture, while bronchoalveolar lavage (BAL) was obtained from intubated and tracheotomized patients for culture and antibiotic susceptibility testing. Swabs of skin and soft tissues were taken, and samples from punctures suspected of infection were sent for culture and antibiotic susceptibility testing. The procedure for collecting these samples was repeated 24 hours after the first sampling. For blood cultures, a set of bottles for aerobic and anaerobic cultivation (bioMérieux, Durham, NC) was used for incubation in the automated blood culture analyzer VIRTUO BACT/ALERT (bioMérieux). The volume of blood taken per bottle was approximately 8 mL, and incubation was performed according to good laboratory practice, following the manufacturer’s recommendations. Urine sampling was performed after urinary catheterization, collecting 15-30 mL of urine. Urine was not kept at room temperature for more than 30 minutes and was stored in the refrigerator for up to 30 minutes if it was not directly inoculated onto the medium. BAL was performed through a flexible bronchoscope. A physiological saline solution of 50-100 mL was injected through the system and immediately aspirated to retain microorganisms from the lower respiratory tract in the aspirated contents. If a classic BAL could not be obtained, a mini-BAL procedure (with less than 50 mL of saline solution) was performed. The choice of sampling site was based on the location of infiltrates on X-ray or computed tomography (CT) images. The BAL sample contained at least 5-10 mL of aspirated content. All samples were transported within a short period (less than 30 minutes) to the microbiology laboratory to preserve the morphology of leukocytes in the samples. A quantitative culture with Gram staining was performed from each sample. Growth of >10^4 colony-forming units/mL was considered positive for samples obtained by BAL.

Blood sampling and assessment of oxidative stress and antioxidative status biomarker parameters

After the diagnosis of sepsis was established, we collected the first sample of venous blood to determine pro-oxidant parameters: TBARS, NO₂⁻, H₂O₂, and O₂⁻, as well as antioxidant parameters: superoxide dismutase (SOD), catalase (CAT), and reduced glutathione (GSH). The blood sampling procedure was repeated 24 hours after the first sampling. Measured pro-oxidants and antioxidants were compared in defined patient groups. Blood samples from the tubes were centrifuged for 10 minutes at 3000 revolutions per minute. Plasma samples and erythrocyte lysates prepared in labeled Eppendorf Safe-Lock tubes were used for the analysis of oxidative stress parameters. All samples were stored at −80°C until measurement. Plasma samples were used to determine the values of TBARS, NO₂⁻, H₂O₂, and O₂⁻, while erythrocyte lysate samples were used to determine SOD, CAT, and GSH. All analyses related to oxidative stress were performed spectrophotometrically (Shimadzu UV-1800 spectrophotometer). The lipid peroxidation index, TBARS, was determined using a 1% solution of thiobarbituric acid (TBA) in 0.05M sodium hydroxide (NaOH) and measured at 530 nm. The measurement unit for TBARS is µmol/mL plasma. For the determination of H₂O₂, the Pick and Keisari method was used, based on oxidation with phenol red in a reaction catalyzed by peroxidase. After incubation for 10 minutes at room temperature, the concentration of H₂O₂ was measured at a wavelength of 610 nm. The measurement unit for H₂O₂ is nmol/mL plasma. The level of O₂⁻ is measured by a reduction reaction with nitro blue tetrazolium (NBT) and TRIS buffer at a wavelength of 530 nm. The measurement unit for O₂⁻ is nmol/mL plasma. NO₂⁻ is measured indirectly through its nitrite products using the Griess method, which employs solutions of sulfosalicylic acid and N-(1-naphthyl)ethylenediamine, measuring the resulting pink product at 550 nm. The measurement unit for NO₂⁻ is nmol/mL plasma. The activity of antioxidants: CAT, SOD, and GSH from erythrocyte lysates was determined using the Beutler method. For determining CAT, a H₂O₂ aqueous solution, CAT buffer, and erythrocyte lysate sample were used, with measurement performed at 240 nm. The measurement unit for CAT is U/g hemoglobin (Hgb)*10³. The activity of SOD was measured after mixing erythrocyte lysate with carbonate buffer and then with epinephrine, with measurement performed at 470 nm. The measurement unit for SOD is U/g Hgb*10³. The determination of reduced GSH concentration was based on the oxidation reaction with 5,5′-dithiobis(2-nitrobenzoic acid), with measurement performed at 412 nm. The measurement unit for GSH is nmol/mL red blood cell (RBC).

Regarding blood sample collection for oxidant/antioxidant levels, it should be emphasized that the balance of oxidants and antioxidants can be influenced by several factors related to the sampling process, including the handling and processing of the samples. Hemolysis during blood collection, delays in processing, and exposure to light or heat can alter oxidative stress markers and antioxidant levels. Proper sampling techniques and immediate processing are crucial to minimize these effects and ensure accurate measurements of the oxidant/antioxidant balance.

Statistical analysis 

Statistical analysis was performed using the commercial statistical software SPSS Statistics for Windows, version 18.0 (SPSS Inc., Chicago, IL). Continuous variables were expressed as mean ± standard deviation (±SD) or median, interquartile range (IQR), as appropriate, while categorical variables were presented as absolute numbers or percentages. The normal distribution of data was tested using the Kolmogorov-Smirnov test. The values of continuous variables were compared using the Mann-Whitney U test, Kruskal-Wallis test, ANOVA test, and Z test, while categorical variables were compared using the chi-square (χ²) test or Fisher’s exact test. Statistical significance was defined as p < 0.05.

## Results

A total of 87 subjects were included in the study, categorized into four groups based on microbiological analyses: group I (GP) included 30 patients (34.5%), group II (GN) included 35 patients (40.2%), group III (GP/GN) included three patients (3.4%), and group IV (NC) included 19 patients (21.8%). The study included 53 (60.9%) male and 34 (39.1%) female participants. The oldest patient was 87 years old and belonged to the GP sepsis group, while the youngest was 22 years old and belonged to the GN sepsis group. Of all patients observed, 62 (71.3%) scored a qSOFA score of 2. The results of the Z-test showed a statistically significant difference between the GP/GN group and all other groups for the qSOFA 2 parameter (p = 0.001). The Z-test results also showed a statistically significant difference between the GP/GN group and all other groups among subjects who experienced septic shock (p = 0.001). Out of 87 patients, 71 (81.6%) required mechanical ventilation. The longest duration of mechanical ventilation was 62 days for a patient in the GN group, and the shortest was four days in the GP/GN group. The average duration of mechanical ventilation for all subjects was 7.84 ± 8.89 days. The longest stay in the ICUs lasted 77 days in the GP/GN group, and the shortest stay was two days across all groups. Based on the Kruskal-Wallis H test, we concluded that there was a statistically significant difference in ICU length of stay (LOS) between groups with different types of infections. Additional analysis using the Mann-Whitney U test (U = 179.000; p = 0.005) showed that the GN group spent statistically significantly (p = 0.04) more days in the ICU compared to the NC group of septic patients. The demographic, clinical characteristics, and final outcomes of the observed groups are presented in Table 1.

**Table 1:** Demographic, clinical characteristics, and final outcome of all observed patients SD, standard deviation; SOFA, Sequential Organ Failure Assessment; qSOFA, quick SOFA; MV, mechanical ventilation; ICU LOS, length of stay at intensive care unit; n, number of respondents; GP, Gram-positive; GN, Gram-negative; NC, negative culture; ‡, Pearson’s chi-square tests; •, Kuskal Wallis test; †, Fisher’s exact test; ″, Z test; ***, Mann-Whitney U test

Variable	Group GP (n = 30; 34.5%)	Group GN (n = 35; 40.2%)	Group GP/GN (n = 3; 3.4%)	Group NC (n = 19;21.8%)	p-value	All patients (n)
Age (±SD)	60.57 ± 15.07	64 ± 14.76	51 ± 20.89	63.83 ± 13	0.38•	62.31 ± 14.70
Sex						
Male	18 (33.96%)	19 (54.3%)	2 (66.7%)	14 (73.7%)	0.57†	53 (60.9%)
Female	12 (40%)	16 (45.7%)	1 (33.3%)	5 (26.3%)		34 (39.1%)
Score						
SOFA (±SD)	7 ± 3.05	6.97 ± 3.27	7.33 ± 4.62	7.16 ± 2.58	0.80•	87 (100%)
qSOFA 2 points (%)	20 (32.26%)	25 (40.32%)	3 (4.84%)	14 (22.58%)	0.001‡ "	62 (71.3%)
qSOFA 3 points (%)	10 (40%)	10 (40%)	0 (0%)	5 (20%)	0.36‡	25 (28.7%)
Clinical characteristics						
Vasopressors (N/%)	13 (32.5%)	18 (45%)	0 (0%)	9 (22.5%)	0,21‡	40 (45.97%)
Septic shock (N/%)	18 (36%)	19 (38%)	1 (2%)	12 (24%)	0.001‡ "	50 (57.5%)
MV duration (±SD)	7.79 ± 7.40	9.53 ± 11.28	2.33 ± 1.53	5.31 ± 4.5	0.11•	71 (81.6%)
ICU LOS (±SD)	10.87 ± 10.44	16.51 ± 16.61	27.67 ± 42.74	7.47 ± 7.21	0.04• ***	87 (100%)
Outcome (28th days)						
Survival (N/%)	18 (36.8 %)	18 (48.6 %)	1 (33.3%)	12 (63.2%)	0.69 †	49 (56.3%)
Non-survival (N/%)	12 (63.2%)	17 (51.4 %)	2 (66.7 %)	7 (36.8%)		38 (43.7%)

Table 2 shows the pro-oxidant values in the analyzed patient groups. A comparison of the median ΔO₂⁻ values among the patient groups, based on the Kruskal-Wallis test, indicated a statistically significant increase in the GN group of septic patients (chi-square test, χ² = 9.327, p = 0.02).

**Table 2:** Biochemical parameters of pro-oxidants in different groups of septic patients IQR, interquartile range; SD, standard deviation; TBARS, thiobarbituric acid reactive substances; H₂O₂, hydrogen peroxide; O₂⁻, superoxide anion radical; NO₂⁻, nitrite; Δ, delta (the difference between the first and second day); n, number of respondents; GP, Gram-positive; GN, Gram-negative; NC, negative culture; *, one-way ANOVA test; **, Kruskal-Wallis test

Variable	Group GP (n = 30; 34.5%)	Group GN (n = 35; 40.2%)	Group GP/GN (n = 3; 3.4%)	Group NC (n = 19; 21.8%)	p-value
TBARS (median, IQR) (µmol/mL)					
Day 1 TBARS	2.58 (0.14)	2.59 (0.15)	2.52	2.59 (0.21)	0.81^**^
Day 2 TBARS	2.64 (0.13)	2.68 (0.19)	2.74	2.71 (0.17)	0.62^**^
Δ TBARS	-0.12 (0.19)	-0.13 (0.32)	-0.22	-0.10 (0.30)	0.68^*^
NO_2_^-^ (median, IQR)(nmol/mL)					
Day 1 NO_2_^-^	3.15 (0.38)	3.17 (0.26)	3.26	3.17 (0.25)	0.55^*^
Day 2 NO_2_^-^	3.24 (0.25)	3.26 (0.13)	3.22	3.19 (0.20)	0.60^**^
Δ NO_2_^-^	0.0 (1.81)	-0.09 (0.42)	1.32	0.04 (1.16)	0.31^*^
H_2_O_2_ (±SD) (nmol/mL)					
Day 1 H_2_O_2_	3. 15 ± 0.18	3.12 ± 0.19	3.00 ± 0.12	3.11 ± 0.21	0.67^*^
Day 2 H_2_O_2_	3.20 ± 0.19	3.12 ± 0.15	3.18 ± 0.23	3.13 ± 0.22	0.30^*^
Δ H_2_O_2_	-0.05 ± 0.25	0.00 ± 0.26	-0.17 ± 0.27	-0.01 ± 0.34	0.62^*^
O_2_^-^ (median, IQR)(nmol/ml)					
Day 1 O_2_^-^	8.57 (0.99)	8.24 (0.99)	8.9	8.07 (1.32)	0.06^**^
Day 2 O_2_^-^	8.24 (1.32)	8.24 (1.65)	7.91	8.24 (1.16)	0.41^**^
Δ O_2_^-^	0 (1.81)	-0.33 (2.64)	1.32	-0.82 (2.56)	0.02^**^

Table 3 presents the antioxidant values in the analyzed patient groups. None of the observed parameters showed a statistically significant difference among the patients studied.

**Table 3:** Biochemical parameters of antioxidants in different groups of septic patients IQR, interquartile range; SD, standard deviation; SOD, superoxide-dismutase; CAT, catalase; GSH, reduced glutathione; Δ, delta (the difference between the first and second day); n, number of respondents; GP, Gram-positive; GN, Gram-negative; NC, negative culture; *, one-way ANOVA test; **, Kruskal-Wallis test

Variable	Group GP (n = 30; 34.5%)	Group GN (n = 35; 40.2%)	Group GP/GN (n = 3; 3.4%)	Group NC (n = 19; 21.8%)	p-value
SOD (median, IQR) (U/g Hgb*10^3^)					
Day 1 SOD	24.42 (10.18)	24.42 (8.14)	24.42	24.42 (10.18)	0.24^**^
Day 2 SOD	16.28 (8.14)	24.42 (8.14)	16.28	16.28 (8.14)	0.55^**^
Δ SOD	0 (8.14.)	8.14 (8.14)	8.14	0 (12.21)	0.67^**^
CAT (median, IQR) (U/g Hgb*10^3^)					
Day 1 CAT	4.62 (3.56)	6 (2.75)	6	5.25 (3.13)	0.38^*^
Day 2 CAT	4.5 (3.63)	3.75 (3)	4.25	4 (3.06)	0.66^* *^
Δ CAT	0.25 (4)	1.75 (3.25)	0	1 (2.5)	0.07^*^
GSH (median, IQR) (nmol/mL RBC)					
Day 1 GSH	74,977.4 (5457.63)	75,367.23 (6237.29)	74,847.46	73,807.91 (4353.11)	0.43^**^
Day 2 GSH	72,508.47 (3313.55)	71,988.70 (4158.19)	69,909.60	73,288.14 (3248.58)	0.40^**^
Δ GSH	2728.81 (7146.89)	2598.87 (5457.63)	4158.20 (0)	1039.54 (7471.75)	0.26^**^

## Discussion

The main finding of this study highlights that no causal relationship was established between measured pro-oxidant and antioxidant values in differentiating the causative agent of sepsis or determining treatment outcomes.

In the GP sepsis group, a positive trend was noted for TBARS, NO₂⁻, and H₂O₂ parameters, with a negative trend for O₂⁻; however, none of these changes were statistically significant. Similarly, in the GP sepsis group, values for the studied antioxidants (SOD, CAT, and GSH) showed a downward trend on the second day, also without statistical significance. In the GN group of septic patients, an increase in pro-oxidant values for TBARS and NO₂⁻ was measured on the second day compared to the first, while O₂⁻ and H₂O₂ values remained constant between the two days, without statistical significance. Additionally, a decrease in antioxidant values for CAT and GSH was measured on the second day in the GN group, while SOD values remained the same on both days without statistical significance. Polymicrobial infection is a scenario that should be considered. Studies have shown that polymicrobial infection is a risk factor for severe sepsis [18].

A specific feature of this study was the occurrence of mixed infections observed in three patients (3.4%). In the present study, no pathogenic microorganism was identified in 19 patients (21.8%), with these septic patients designated as the NC group. According to the available literature, one-third of sepsis patients do not have an identified pathogen [19]. A negative bacterial culture in patients could be explained by the timely initiation of antimicrobial therapy (resulting in a negative culture). Some studies have included only patients with a single positive culture, while others included patients with polymicrobial infections. Research by Mihaljevic et al. (2023) found elevated oxidative stress levels and significantly reduced antioxidant activity, particularly SOD, in critically ill septic patients compared to a control group [20]. In their study, Takeda et al. discovered increased TBARS levels in septic patients, indicating elevated lipid peroxidation [21]. Reduced antioxidant levels were also detected [22]. These data partially align with our results. According to Tang et al. (2023), a meta-analysis including Chinese and English observational studies found no significant difference in survival rates, hospital stay duration, or SOFA scores between sepsis cases caused by GN and GP bacteria. This meta-analysis also showed a higher incidence of septic shock in the GN group compared to the GP group of septic patients [23].

A retrospective study using online data on septic patients hospitalized at a tertiary academic medical center in Boston reported a higher 28-day mortality rate for GP infections compared to GN infections [24]. A study conducted in China from 2013 to 2020 revealed that mixed infections (GP/GN) were more severe than monomicrobial GN or GP bacterial infections, more likely to cause organ dysfunction, and carried a higher risk of death. There was no difference between monomicrobial GN and GP bacterial infections [25]. These findings partially align with our study results. Examining the age and gender distribution of patients shows that the average patient age was 62.31 years, with a higher prevalence of male septic patients compared to female participants. A four-year study by Juliana et al. found that sepsis prevalence was significantly higher among patients aged 50-69, with males showing a higher prevalence than females. This suggests that this age group requires targeted attention and preventive strategies to reduce sepsis incidence. A brief literature review on the reasons for higher sepsis incidence in men suggests that the causes are likely multifactorial, with behavioral, physiological, and immunological factors likely playing a role [26]. Studies by Santella et al. and Vasudeva et al. confirm these findings [27,28]. Our results are consistent with these authors’ findings. In this study, of the 87 patients included, GP sepsis was diagnosed in 30 patients (34.5%) and GN sepsis in 35 patients (40.2%).

According to a 24-hour prevalence study conducted in 1,150 centers across 88 countries in 2017, 5,259 of 15,202 participants had at least one positive microbiological culture, of which 67% were GN bacteria, 37% were GP bacteria, and 16% were fungal infections [29]. The Sepsis Occurrence in Acutely Ill Patients (SOAP) study report, published in 2006 and conducted in ICU units across 24 European countries, showed a roughly equal distribution of GP and GN isolates among sepsis patients [30]. Martin et al. analyzed the most common isolates in U.S. hospitals from 1979 to 2000, finding that GN bacteria predominated until 1987 [30]. GP isolates increased annually, becoming the dominant isolates by 2000 [30]. Our research findings partially align with these literature sources. This study recorded a statistically significant difference in ICU LOS. The GN septic patient group had a longer ICU stay than the GP group (p = 0.045), aligning partially with other literature sources. In a retrospective study by Guo et al., examining septic patients hospitalized in a tertiary academic medical center in Boston, the GN group had a longer ICU stay than the GP group [24].

This study is limited by the insufficient number of participants and by being conducted at a single medical center. Ideal conditions for this type of research would involve measuring oxidant and antioxidant levels for each patient before the onset of sepsis, allowing comparison with redox status during sepsis. However, this was not feasible due to the difficulty of predicting which patients would develop an inflammatory response.

## Conclusions

In this study, based on the measured values of pro-oxidants and antioxidants, it is evident that there is no predictive significance for the final treatment outcome and the type of causative agent of sepsis. The pathophysiological mechanisms of sepsis caused by GP and GN bacteria differ significantly, and the role of antioxidants and pro-oxidants remains insufficiently studied. Further research is needed in this regard.
